# Strange Case of a Sojourn in Saranac

**DOI:** 10.3201/eid2703.AC2703

**Published:** 2021-03

**Authors:** Terence Chorba

**Affiliations:** Centers for Disease Control and Prevention, Atlanta, Georgia, USA

**Keywords:** art science connection, emerging infectious diseases, art and medicine, about the cover, strange case of a sojourn in Saranac, public health, tuberculosis, tuberculosis and other mycobacteria, bacteria, Mycobacterium tuberculosis, respiratory infections, bronchiectasis, history, literature, sculpture, Robert Louis Stevenson, Gutzon Borglum, Edward Trudeau

**Figure 3:**
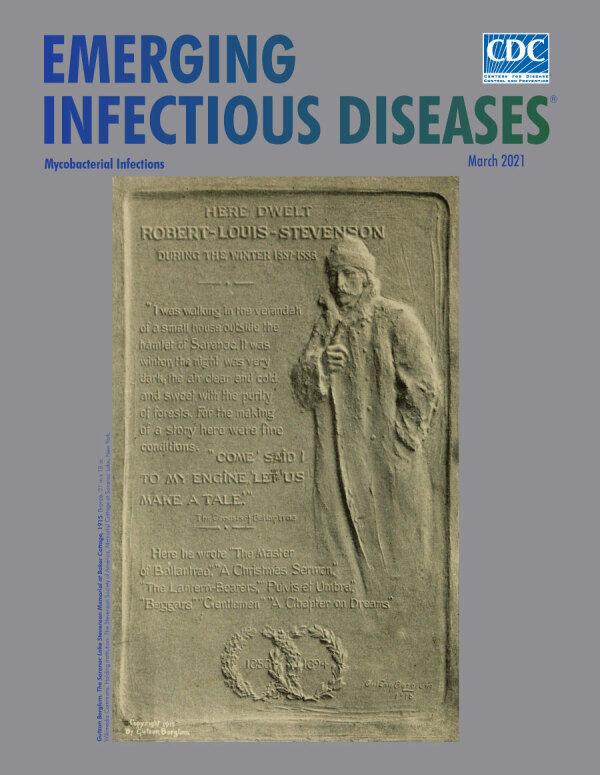
**Gutzon Borglum. The Saranac Lake Stevenson Memorial at Baker Cottage, 1915.** Bronze. 31 in x 18 in. From Wikimedia Commons. Holding institution: The Stevenson Society of America, Memorial Cottage at Saranac Lake, New York.

Robert Louis Stevenson, the renowned Scottish author and poet, was born in 1850 in Edinburgh, Scotland. From childhood onward, he suffered from frequent chest infections, fevers, and hemoptysis. This master who gave us *Treasure Island* (1881–83), *A Child’s Garden of Verses* (1885), *Kidnapped* (1886), and *Strange Case of Dr Jekyll and Mr Hyde* (1886) lived his brief 44 years in an era when tuberculosis (then called consumption) was widespread in Europe but laboratory and radiologic diagnostics were not available.

On March 24, 1882, the date on which World TB Day is based, Robert Koch announced his discovery of the causative organism of tuberculosis, Mycobacterium tuberculosis*. Through Koch’s use of* alkalinized methylene blue stain and a brown counterstain for contrast, this discovery laid to rest the theory that tuberculosis was congenital, a belief stemming from its extensive occurrence in families. In 1895, Wilhelm Röntgen first described the potential medical application of radiography when he captured the image of the bony and soft-tissue structures of his wife’s hand on a photographic plate. Without bacteriology or radiography, Stevenson’s persistently cachectic body habitus and pulmonary symptoms were thought to be consistent with tuberculosis, although other diagnoses have been proposed. These have included chronic idiopathic bronchiectasis, sarcoidosis, and hereditary hemorrhagic telangiectasia, any of which could potentially have been exacerbated by residual scarring from the bouts of pneumonia he experienced as a child and by his chain-smoking as an adult. 

By 1887, Stevenson had achieved considerable wealth from his writings and acted on medical advice to seek a climate drier than that of the British Isles. He and his family set out for Colorado, but upon arriving in Massachusetts, he learned of the growing reputation of physician Edward Livingston Trudeau, who founded the Adirondack Cottage Sanatorium at Saranac Lake, New York, for the cure of pulmonary tuberculosis. In October 1887, Stevenson, together with his wife, his mother, his stepson, and a family servant, moved into Baker Cottage, a house adjacent to the sanatorium. Stevenson remained there through the winter and produced many essays for *Scribner’s Magazine* and completed most of a novel, *The Master of Ballantrae.* Trudeau was unable to demonstrate the presence of tubercle bacilli in Stevenson’s sputum. Stevenson left the sanatorium in April 1888 and travelled to San Francisco, Hawaii, and the Samoan Islands, where he died in 1894. 

**Figure 1:**
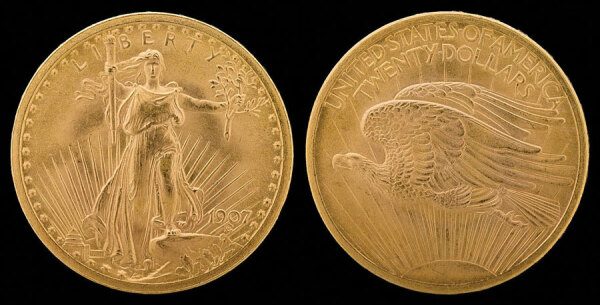
Double Eagle, US $20 gold (fineness 0.9000) coin, 34 mm, 33.436 g, designed by Augustus Saint-Gaudens, 1907. Obverse: Bas relief representation of Liberty, personified by a tall, robed woman striding forward, bearing a torch in her right hand and an olive branch in her left hand. Rays of a sunrise in the background. Reverse: Young eagle in flight, silhouetted by the rays of a sunrise. National Numismatic Collection, National Museum of American History, Washington, DC, USA. Photograph by Jaclyn Nash.

**Figure 2:**
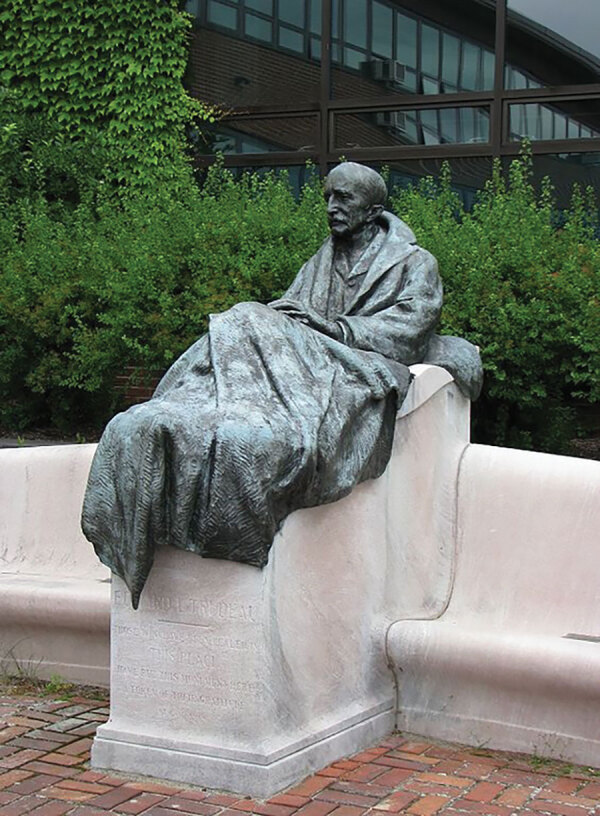
Gutzon Borglum. Statue of E.L. Trudeau, 1918. Bronze. From Historic Saranac Lake Wiki. Originally erected at the Adirondack Cottage Sanatorium. Moved to the Trudeau Institute, Saranac Lake, New York, 1964. The statue is a representation of E. L. Trudeau as a male tuberculosis patient with a blanket folded on his lap. In the pedestal is an inscription with a folk-saying favorite of Trudeau, *“Guerir quelquefois, soulager souvent, consoler toujours*” (To cure sometimes, to relieve often, to comfort always). Trudeau started the facility, which has been credited with being the first sanatorium in the United States, at Saranac Lake in 1885, at the dawn of the nation’s sanatorium movement, which peaked with over 108,000 beds in 1954. Sanatoriums remained the standard of care for tuberculosis for over half a century in the United States; after streptomycin was discovered in 1943 and antimicrobial drugs were subsequently used to treat and cure tuberculosis, sanatoriums closed or were transformed into general hospitals.

In 1915, the Saranac Chapter of the Stevenson Society commissioned John Gutzon Borglum, an American sculptor, to create the bronze bas-relief featured on the cover of this month’s journal, to adorn the wall of Baker Cottage in memory of Stevenson. Relief is the term used to describe sculptural images that remain attached to a solid background plane of the material out of which the images themselves have been sculpted. Bas-relief is relief in which images of faces or figures are rendered with less depth than they possess in life and in which no aspect of the image is cut away from the underside. The master of this technique in America was Augustus Saint-Gaudens (1848–1907), an inspiration to Borglum and fellow alumnus of the École des Beaux-Arts in Paris. Saint-Gaudens created the exquisite $20 US gold coin with the standing female figure of Liberty set in bas-relief, minted from 1907 through 1933 (Figure 1). Of the Saranac bas-relief, the *New York Times* article from the day of the unveiling described the intent of the artist: “[Stevenson is] shown walking on the veranda where…he says in his letters he gained inspiration for Ballantrae and the great Scribner essays. The figure, in fur cap and coat, a thin hand clutching the collar together in the face of the zero blast, breathes the spirit of the sick man out of doors, and the lift of the head as he says ‘Come, let us make a tale!’ reveals the rare spirit of the heroic in physical adversity.” After Edward Trudeau died in 1915, Borglum was also commissioned to craft a life-size statue of the founder for the Saranac sanatorium (Figure 2). 

Borglum was born to Mormons in Idaho in 1867, but grew up in Nebraska and Kansas, where he began his formal art training. At age 22, he enrolled at the Académie Julian and the École des Beaux-Arts, where sculptor Auguste Rodin (1840–1917) was one of his instructors. Over the next several years, Borglum developed more skills and success as a sculptor in Spain and England. In 1901, he embarked on a prolific period in America; his works include the equestrian bronze in Sheridan Circle in Washington, DC, some of the statuary of the Cathedral of St. John the Divine in New York City, a memorial to the fallen North Carolina infantry from Pickett’s Charge at Gettysburg, and a bust of Abraham Lincoln now in the United States Capitol crypt. He had white supremacist leanings, and in 1915, the same year he made the Saranac bronze bas-relief, he also was hired to begin a granite bas-relief carving to honor the Confederacy on Stone Mountain, Georgia. In 1925, controversy with the committee who gave him the commission ended with his being fired and his work being blasted off the mountain's face, but the experience gave him knowledge of the methods for sculpting on a scale that made possible his subsequent creation of the national monument at Mount Rushmore. From 1927 until his death in 1941, Borglum labored on that project, his greatest opus. Carved into granite in the Black Hills of South Dakota on land that had been illegally seized from the Lakota tribe during a gold rush in the 1870s, this national memorial has also been the subject of criticism for aspects of the actions of each of the men it memorializes. Even there, a bas-relief quality informs the work, especially in the image of Abraham Lincoln, rising out from within the rock toward the viewer. 

The Saranac bas-relief was a piece of genius for Borglum. Stevenson emerges from the flat plane of the bronze to convey the image of *spes phthisica*, a storied term used in the nineteenth century to refer to an elated mental state thought to be experienced by creative people with consumption, which inspired them to produce great works.
